# Supplementary material for: A Longitudinal Study of Hematology and Stress Biomarker Profiles in Young Asian Elephants (Elephas Maximus) in Relation to Elephant Endotheliotropic Herpesvirus (EEHV) in Thailand
Source: Animals (Basel). 2021 Aug 28;11(9):2530. doi: 10.3390/ani11092530 (PMC8471699; doi:10.3390/ani11092530)
Supplement: Supplementary file 1 [file animals-11-02530-s001.zip › animals-1345747-supplementary.pdf]

Season	ID	Groups	Status	PCV (%)	RBC (x 106 cells/ul)	WBC (cells/ul)	Heterophil (cells/ul)	Lymphocyte (cells/ul)
Winter	E1	Normal value	Prior EEHV	43.80	3.46	20116.20	7587.40	11869.80
Winter	E2	Normal value	Non EEHV	35.00	2.99	19364.00	5051.20	11978.20
Winter	E3	Normal value	Prior EEHV	39.60	3.32	17274.00	2920.80	10703.20
Winter	E4	Normal value	Prior EEHV	35.40	2.84	16322.00	4482.00	7137.40
Winter	E5	EEHV-HD	Prior EEHV	32.50	3.05	19232.50	5819.25	9833.25
Winter	E6	Normal value	Prior EEHV	38.00	3.12	16214.00	3453.00	9166.60
Winter	E7	Normal value	Prior EEHV	37.38	3.13	18087.12	4885.61	10114.74
Winter	E8	Normal value	Non EEHV	37.38	3.13	18087.12	4885.61	10114.74
Winter	E9	Normal value	Non EEHV	37.80	3.04	21622.00	3685.00	13980.20
Summer	E1	Normal value	Prior EEHV	43.00	3.47	15920.00	4367.00	8293.00
Summer	E2	Normal value	Non EEHV	32.25	2.81	21990.00	4316.75	13421.50
Summer	E3	Normal value	Prior EEHV	37.68	3.11	19844.00	4122.92	11898.23
Summer	E4	Normal value	Prior EEHV	36.25	2.89	14810.00	5558.75	6918.00
Summer	E5	EEHV-HD	Prior EEHV	25.25	2.31	11130.00	2978.00	6207.75
Summer	E6	Normal value	Prior EEHV	36.25	2.97	18330.00	5230.00	7116.75
Summer	E7	Normal value	Prior EEHV	33.54	2.81	17220.80	4441.28	9112.45
Summer	E8	Normal value	Non EEHV	33.79	2.82	16266.96	4466.19	8250.64
Summer	E9	Normal value	Non EEHV	33.02	2.76	15551.55	4534.84	7521.12
Rainy	E1	Normal value	Prior EEHV	40.25	3.17	17157.50	3486.75	9989.50
Rainy	E2	Normal value	Non EEHV	33.25	2.71	17747.50	4033.50	10383.50
Rainy	E3	Normal value	Prior EEHV	35.51	2.88	16818.85	4018.36	9298.04
Rainy	E4	Normal value	Prior EEHV	36.34	2.92	17241.28	3846.20	9890.35
Rainy	E5	EEHV-HD	Prior EEHV	NA	NA	NA	NA	NA
Rainy	E6	Normal value	Prior EEHV	36.34	2.92	17241.28	3846.20	9890.35
Rainy	E7	Normal value	Prior EEHV	35.36	2.86	17262.23	3936.07	9865.56
Rainy	E8	Normal value	Non EEHV	35.88	2.89	17140.91	3911.71	9736.07
Rainy	E9	Normal value	Non EEHV	35.25	2.86	17597.50	3746.00	10618.00

Monocyte (cells/ul)	Eosinophil (cells/ul)	Platelet (x 103 cells/ul)	M:H ratio	SC (ng/ml)	FGM (ng/ml)	SIgA (µg/ml)	FIgA (µg/ml)
3137.60	295.80	553.40	0.41	1.66	23.55	1.41	2.10
5578.80	348.80	427.60	1.10	0.79	33.35	5.89	4.63
3415.60	198.00	428.60	1.17	0.94	27.24	2.49	2.37
4599.20	103.60	453.20	1.03	1.95	33.36	2.12	1.95
3218.00	362.00	602.75	0.55	1.07	52.13	4.81	6.13
3093.60	501.00	374.00	0.90	1.54	23.92	1.28	2.30
3840.47	301.53	473.26	0.79	1.32	32.26	3.00	3.25
3840.47	301.53	473.26	0.79	1.32	32.26	3.00	3.25
3796.00	161.00	429.80	1.03	0.92	53.51	5.13	4.36
2694.50	535.25	553.00	0.62	1.49	32.63	2.78	3.19
3173.00	219.25	469.00	0.74	0.65	45.81	5.07	9.04
3221.17	305.17	483.93	0.78	1.02	43.99	4.33	5.53
2316.00	0.00	433.75	0.42	1.33	51.42	3.09	3.56
1838.50	106.00	394.75	0.62	0.42	41.85	2.50	2.62
3453.75	413.50	398.00	0.66	1.55	37.41	4.08	2.32
2800.48	208.78	435.89	0.63	0.99	44.09	3.81	4.61
2725.98	206.69	429.26	0.61	1.06	43.75	3.56	3.73
2626.94	186.99	418.33	0.58	1.07	43.70	3.41	3.37
3346.00	291.00	550.75	0.96	1.05	37.72	3.71	2.01
2698.25	395.00	471.75	0.67	0.72	29.07	12.60	1.33
2890.40	291.00	480.28	0.72	0.95	36.83	6.57	2.24
2978.22	325.67	500.93	0.77	0.91	34.54	7.63	1.86
NA	NA	NA	NA	NA	NA	NA	NA
2978.22	325.67	500.93	0.77	0.91	34.54	7.63	1.86
2886.27	334.33	488.47	0.73	0.87	33.75	8.61	1.82
2933.27	319.17	492.65	0.75	0.91	34.91	7.61	1.94
2909.25	276.75	401.00	0.78	0.73	25.48	3.19	1.81
